# Synthesis of Phosphorus-Containing Polyanilines by Electrochemical Copolymerization

**DOI:** 10.3390/polym12051029

**Published:** 2020-05-01

**Authors:** Beatriz Martínez-Sánchez, Andrés Felipe Quintero-Jaime, Francisco Huerta, Diego Cazorla-Amorós, Emilia Morallón

**Affiliations:** 1 Departamento de Química Física and Instituto Universitario de Materiales de Alicante (IUMA), University of Alicante, Ap. 99, 03080 Alicante, Spain; beatriz.ms@ua.es (B.M.-S.); andres.quintero@ua.es (A.F.Q.-J.); 2 Departamento de Ingeniería Textil y Papelera, Universitat Politècnica de València, Plaza Ferrándiz y Carbonell, 1. E-03801 Alcoy, Spain; frahuear@txp.upv.es; 3 Departamento de Química Inorgánica and Instituto Universitario de Materiales de Alicante (IUMA), University of Alicante, Ap. 99, 03080 Alicante, Spain

**Keywords:** polyaniline, phosphorus, electrochemical polymerization, modified polyaniline

## Abstract

In this study, the phosphonation of a polyaniline (PANI) backbone was achieved in an acid medium by electrochemical methods using aminophenylphosphonic (APPA) monomers. This was done through the electrochemical copolymerization of aniline with either 2- or 4-aminophenylphosphonic acid. Stable, electroactive polymers were obtained after the oxidation of the monomers up to 1.35 V (reversible hydrogen electrode, RHE). X-ray photoelectron spectroscopy (XPS) results revealed that the position of the phosphonic group in the aromatic ring of the monomer affected the amount of phosphorus incorporated into the copolymer. In addition, the redox transitions of the copolymers were examined by in situ Fourier-transform infrared (FTIR) spectroscopy, and it was concluded that their electroactive structures were analogous to those of PANI. From the APPA monomers it was possible to synthesize, in a controlled manner, polymeric materials with significant amounts of phosphorus in their structure through copolymerization with PANI.

## 1. Introduction

The development of new multifunctional materials with tunable properties has received increasing attention in many research fields in recent years. This is because of their outstanding mechanical, optical, magnetic, and electrical properties, as well as their low cost and easiness of preparation. As such, conducting polymers have proven to be promising materials for a number of technological applications [1,2]. Among these materials, polyaniline (PANI) stands out as an environmentally stable polymer [3,4] that can be used in fuel cells [5], energy storage systems [6,7], sensors [8], or, more recently, as a precursor of nitrogen-containing carbon materials with exceptional electrocatalytic behavior [9,10]. Interestingly, its electrical properties and structure can be tailored by appropriately selecting the acid doping conditions [11]. Nevertheless, PANI has some major disadvantages, including a lack of solubility in common solvents and a narrow operation pH range in its conducting state [4,12], hindering its industrial applications.

In this sense, the combination of PANI with highly potential materials such as metallic nanoparticles (e.g., Co and Fe) [13], corrosion inhibitors (organic acids dopants) [14], or carbon nanomaterials [15], results in hybrid nanocomposites with enhanced properties. However, a simpler and promising alternative route is the chemical functionalization of PANI with carboxylic [16,17], sulfonic [18,19,20], nitrate [21], alkyl [22,23], or alcohol [24,25,26] groups, among others, which is usually carried out to overcome these problems. Indeed, copolymerization and post-functionalization are the most extended synthesis routes to obtain modified polyanilines. This is because, in many cases, steric and/or inductive effects make the homopolymerization of substituted monomers difficult to accomplish and, in others, the resulting fully-functionalized, short-chain oligomeric products are highly soluble and difficult to isolate. The functionalization of PANI with phosphonic groups is particularly remarkable because, in contrast to sulfonic or carboxylic derivatives, two acidic protons are available. In this way, the first proton can participate in acid doping, whereas the second can provide additional properties such as acid/base complexation, extra resistance to neutralization, or higher proton conductivity, as required in polymer membranes for specific applications. Phosphorus species could be interesting for the preparation of hybrid materials such as polymer/metal nanoparticles composites, because the interaction with the metallic precursor could be improved. Moreover, the use of polyaniline-like polymers and copolymers with different functional groups is being studied to obtain different materials such as biosensors and functionalized carbon for applications in electrocatalysis, batteries, etc. [27,28,29].

Nonetheless, few works can be found regarding phosphonated polyanilines, and most of them use chemical routes to achieve functionalization, either on the rings or on secondary nitrogen atoms. It was reported that the chemical post-modification of polyaniline could be attained by direct phosphonation under specific experimental conditions [30,31,32,33]. Alternatively, the chemical oxidation of phosphonated methoxy- or benzyl- aniline monomers yielded polyaniline-derived structures showing additional functionalities to the phosphonic group [34,35]. However, to the best of our knowledge, the electrochemical incorporation of C-PO(OH)_2_ groups to polyaniline has not yet been carried out. In the present work, the electrochemical synthesis of phosphonic ring-substituted polyanilines will be carried out following two different strategies. First, the homopolymerization of *ortho-* and *para*-aminophenylphosphonic acids isomers will be analyzed. Second, copolymerization with aniline will be investigated in order to reduce the high C-PO(OH)_2_ substitution level obtained from homopolymerization. The aim is to obtain a self-doping material showing a chemical structure as similar as possible to the well-known carboxylated or sulfonated polyanilines. Attention will be paid to the spectroelectrochemical response of the phosphonated structure within the potential region of the transition between oxidation states. The synthesized materials will be extensively characterized by X-ray photoelectron spectroscopy (XPS), cyclic voltammetry (CV), and in situ Fourier-transform infrared (FTIR) spectroscopy.

## 2. Experiments

The phosphonated monomers employed were 2- and 4-aminophenylphosphonic acid (2-APPA and 4-APPA, respectively) with 95% purity, supplied by Chem Space (Riga, Lithuania). The chemical structure of both monomers is displayed in Figure S1. Aniline ACS reagent (99.5%) was obtained from Sigma-Aldrich (Merck, Darmstadt, Germany). Prior to use, distillation in vacuum was carried out in order remove oligomeric products generated during storage. Perchloric acid (HClO_4_, 70%) was employed as background electrolyte and was purchased from Merck. All aqueous solutions were prepared with ultrapure water (18.2 MΩ·cm, Millipore^®^ Milli-Q^®^ water, Merck, Darmstadt, Germany).

Prior to the electrooxidation of monomers, the working polycrystalline platinum electrode, with a geometric area of 17.2 mm^2^, was submitted to a thermal treatment and subsequently protected from the laboratory atmosphere with a droplet of ultrapure water until use. Electrochemical syntheses and characterizations were carried out by cyclic voltammetry using an eDAQ Model EA163 potentiostat coupled to an EG&G Parc Model 175 wave generator, whereas data acquisition was performed with an EDAQ e-coder 410 unit (eDAQ EChart data acquisition software, Warszawa, Poland). The electrochemical setup was a standard 3-electrode cell, where a reversible hydrogen electrode (RHE) immersed in the working solution was employed as the reference electrode. A platinum wire was used as counter electrode (CE). The working electrode was immersed in the working solutions at low potential (0.2 V) to avoid the initial oxidation of monomers.

In situ Fourier-transform infrared (FTIR) spectroscopy experiments were carried out in a Nicolet 5700 spectrometer equipped with a mercury cadmium telluride (MCT) detector cooled with liquid nitrogen. A mirror platinum disk electrode of 10 mm in diameter was employed as the working electrode in these experiments. Moreover, the spectroelectrochemical cell was provided with a prismatic CaF_2_ window beveled at 60°, against which the working electrode was pressed to conform the thin-layer configuration. FTIR spectra were expressed in the usual form as the difference between the sample spectrum (collected at the sample potential) and the reference spectrum (collected at the reference potential): ΔR/R. In these conditions, the negatively oriented absorption bands (downwards) were displayed when vibrational modes appeared or intensified at the sample potential. On the contrary, positively oriented absorption bands (upwards) were related to species that disappeared or became IR-inactive at the sample potential.

X-ray photoelectron spectroscopy (XPS) experiments were carried out in a VG-Microtech Multilab 3000 spectrometer (VG Scientific, Sussex, UK) equipped with a semispherical electron analyzer with 9 channeltrons (passing energy of 2–200 eV) and an X-ray source with Al radiation (Kα 1253.6 eV). The deconvolutions of the P2p and N1s peaks were performed by minimum squares fitting using Gaussian–Lorentzian curves, while the Shirley method was used for background determination. The P2p spectra were analyzed considering the spin-orbit splitting into P2p3/2 and P2p1/2 with a 2:1 peak area ratio and 0.87 eV splitting [36,37].

## 3. Results and Discussion

### 3.1. Electrochemical Homopolymerization of 2-APPA and 4-APPA

**Figure 1:**
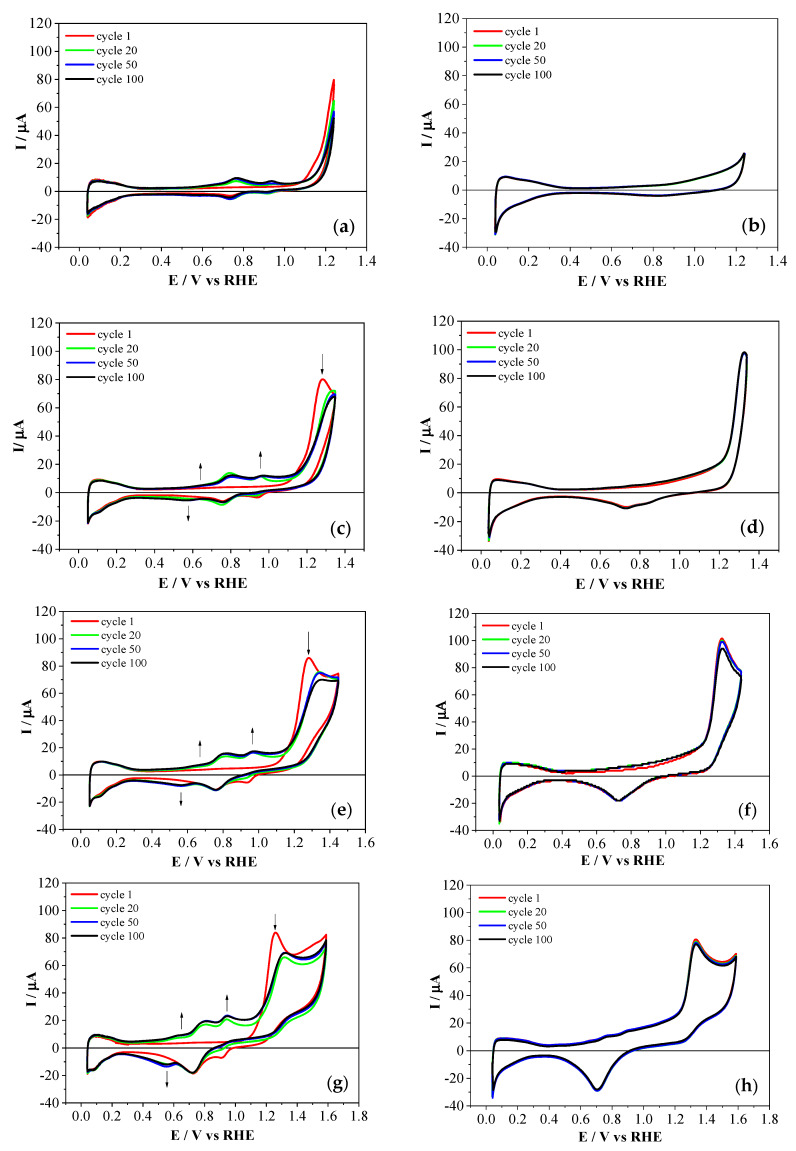
Cyclic voltammograms obtained during the 1st, 20th, 50th, and 100th cycles in 1 M HClO_4_ on the polycrystalline Pt electrode in the presence of 1 mM 2-aminophenylphosphonic acid (2-APPA, left) and 1 mM 4-aminophenylphosphonic acid (4-APPA, right), at 50 mV·s^−1^ under N_2_ atmosphere at different upper potential limits: (**a**,**b**) 1.25 V, (**c**,**d**) 1.35 V, (**e**,**f**) 1.45 V, and (**g**,**h**) 1.60 V. RHE: reversible hydrogen electrode.

Figure 1 shows cyclic voltammograms obtained during the 1st, 20th, 50th, and 100th cycles in presence of 2-APPA (Figure 1a,c,e,g) and 4-APPA (Figure 1b,d,f,h) at different upper potential limits (1.25 V, 1.35 V, 1.45 V, and 1.60 V) for a platinum electrode, based on a previous study of stepwise upper potential limit (See Figure S2).

It was observed that the hydrogen adsorption/desorption processes that occurred at the platinum electrode between 0.05 V and 0.45 V (see Figure S3) were inhibited by the adsorption of monomers on the surface. A further increase in the upper potential limit at around 1.20 V generated an irreversible anodic process in all the voltammograms, related to the oxidation of either 2-APPA or 4-APPA. Interestingly, both the onset potential for oxidation and the peak potential showed an important dependence on the position of the substituent at the aromatic ring, showing onset potentials of 1.05 V and 1.15 V and values of peak potential of 1.28 V and 1.33 V for 2-APPA and 4-APPA, respectively, with the oxidation of the substituted-aniline with the phosphonic moiety in the *ortho*-position being more favorable. This behavior might be a consequence of an effect of the substituent position, which facilitates the oxidation of the amine to form radical species as the initial step of the polymerization.

Additionally, in the case of 2-APPA, during the scan to less positive potential two reduction peaks were clearly observed. The peak appearing at approximately 0.75 V could be associated with the platinum oxide reduction process, whereas the one at 0.94 V could be associated with the reduction of products generated during the oxidation of the monomer. These processes are clearly observed in Figure 1c. Meanwhile, the oxidation peak showed a decrease in the current density upon cycling. When the positive potential was higher than 1.35 V, this oxidation peak shifted to higher positive potentials after the first cycle. Furthermore, two redox processes of less intensity at 0.97/0.94 V and 0.63/0.58 V were formed after electrochemical oxidation and their current increased with cycling, similarly to the PANI processes in acid media (See Figure S4), suggesting that 2-APPA oxidation promoted the formation of active species such as dimers and/or short-chain oligomers for all the upper potential limits studied (Figure 1e,g) and the formation of a thin layer onto the electrode surface. In contrast, during the oxidation of 4-APPA, after the oxidation peak at high potentials no new redox processes were observed on the platinum surface during the scan towards less positive potentials (Figure 1b,d,f,h). In this case, only a platinum oxide reduction peak at 0.73 V (See Figure S3) was observed. Thus, the development of a polymeric film was more greatly inhibited from 4-APPA than from 2-APPA. This result could be a consequence of the blocking effect of the phosphonic group in the *para*-position, which is where the PANI-like polymer would grow. Despite all these facts, it is worth mentioning that there was no significant decrease in the oxidation peak associated with the 4-APPA oxidation with cycling, suggesting that a polymeric film was not formed on the platinum electrode surface [20].

**Figure 2:**
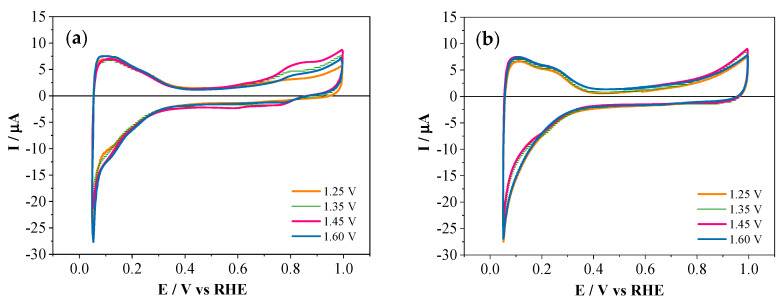
Stable cyclic voltammograms of characterization in 1 M HClO_4_ at 50 mV·s^−1^, obtained after oxidation during 100 cycles of the monomers: (**a**) 1 mM 2-APPA and (**b**) 1 mM 4-APPA under N_2_ at different potentials.

To study the possible formation of oligomers supported on the platinum electrode, once the platinum electrode was oxidized in the presence of APPA isomers at different upper potential limits, electrochemical characterization in an electrochemical cell free of monomer was performed (Figure 2). In both cases, the modified platinum electrode showed a blocking of the hydrogen adsorption/desorption processes that occurred in platinum, confirming the presence of adsorbed species on the electrode surface. In the case of 2-APPA a small redox process was observed in the voltammogram at around 0.80 V, but its current was very low. In the case of 4-APPA, the lack of redox processes suggested that growth of a polymeric layer did not occur on the electrode surface with the oxidation of the phosphonic acid monomers in comparison with the response of the PANI layers at the same conditions (See Figure S5). No important growth of homopolymer occurred with either of the monomers.

Since an increase in the monomer concentration can facilitate the formation of polymer layers on the electrode surface, similar experiments with a 10-times higher concentration of monomer were performed (Figure S6). The results showed that a higher concentration produced an increase in the current of the oxidation peak at high potential once the electrooxidation took place. However, the electrochemical characterization in the absence of monomer (Figure S6c) showed no important growth or development of the electrochemical redox processes, even in acid media, thus confirming the formation of either a very thin or unstable polymeric layer.

### 3.2. Electrochemical Copolymerization of 2-APPA and 4-APPA with Aniline

**Figure 3:**
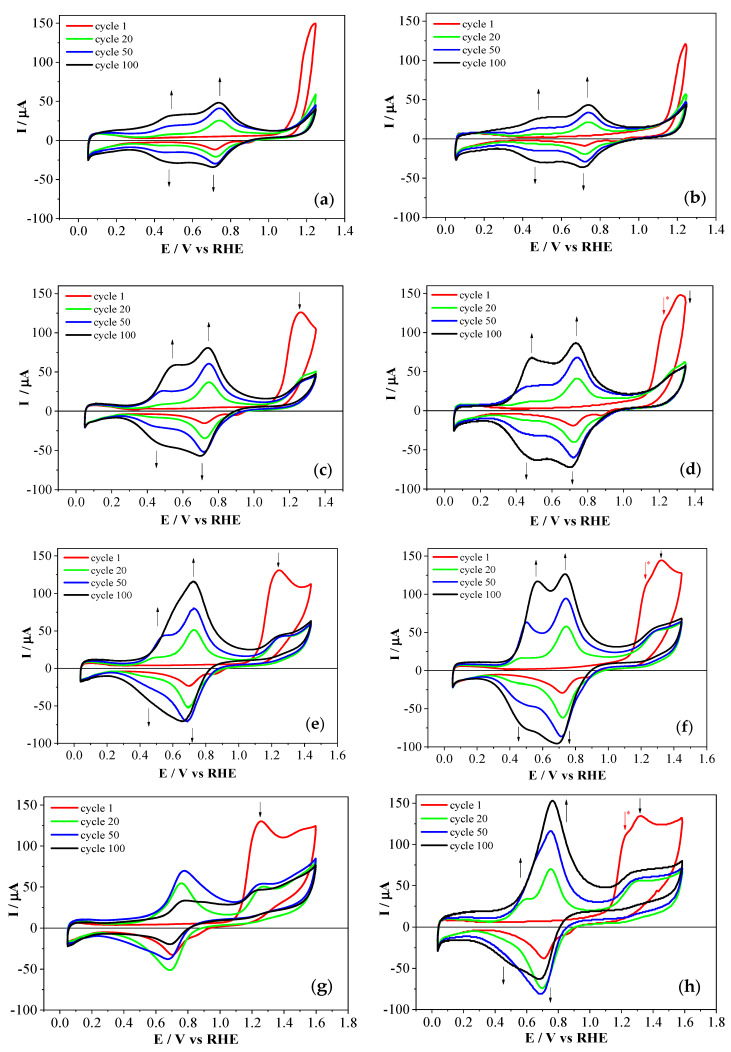
Cyclic voltammograms obtained during the oxidation of aniline and 2-APPA (left) and 4-APPA (right) (monomer concentrations of 1 mM, respectively, at a molar ratio of 1:1) obtained during the 1st, 20th, 50th, and 100th cycles in 1 M HClO_4_ on the polycrystalline Pt electrode, at 50 mV·s^−1^ under N_2_ atmosphere at different upper potential limits: (**a**,**b**) 1.25 V, (**c**,**d**) 1.35 V, (**e**,**f**) 1.45 V, and (**g**,**h**) 1.60 V.

Taking into account that the electrooxidation of phosphorus-modified monomers does not result in the growth of stable polymeric films on Pt, copolymerization with aniline was considered as an alternative strategy to achieve phosphonation. Figure 3 shows cyclic voltammograms obtained during the electrochemical copolymerization of aniline and aminophenylphosphonic acids in a 1:1 molar ratio at different upper potential limits. The images of the platinum electrode surface taken after 100 polymerization cycles are displayed in Figure S7.

The presence of aniline in the electrolyte caused an important increase in the current of the peak associated with monomer oxidation during the first scan and, in addition, the displacement of the onset potential towards less positive values. For PANI–4APPA copolymerization at potentials above 1.25 V (Figure 3d,f,h), a double oxidation peak (marked with a red arrow*) could be clearly observed (around 1.23 V), and was associated with the oxidation of aniline. In subsequent cycles, a progressive decrease in the current was recorded as a result of the formation of a polymeric film on the electrode surface.

Considering all the potentials studied for each monomer, the appearance of two main redox processes at around 0.48/0.45 V and 0.74/0.71 V, which more or less overlapped and were shifted towards more positive potentials as compared to PANI, could be observed. Interestingly, these redox processes showed a dependence on the position of the substituent in the APPA and the upper potential limit applied during electrochemical synthesis. These could be associated with the typical redox processes recorded for PANI synthesized electrochemically in acid medium. The increase in potential produced the overlapping of both redox processes, as can be observed in Figure 3g,h, giving rise to one redox process. This change was a consequence of an over-oxidation of the polymeric film at higher potentials, promoting a change in the chemical nature of the polymer layer. In addition, further degradation of the film or even loss of material could occur in these conditions. In fact, it must be noted that for all the potentials and for both substituted anilines the increase in the number of cycles generated constant growth in the current peaks, associated with the increase in the mass of polymer deposited, except in the case of the PANI–2APPA copolymer at 1.60 V that decreased in cycle 100.

**Figure 4:**
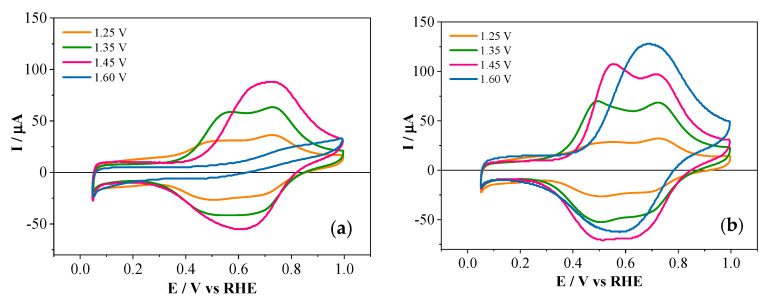
Stable cyclic voltammograms during the characterization of the copolymers in 1 M HClO_4_ at 50 mV·s^−1^, obtained after the electrode modification with the copolymer films (monomer concentration of 1 mM, respectively, at a molar ratio of 1:1): (**a**) PANI–2APPA and (**b**) PANI–4APPA. PANI: polyaniline.

Figure 4 shows the voltammetric characterization of polymeric films in the absence of monomers. The presence of redox processes revealed the formation of electroactive films in all cases. Moreover, the electrochemical response showed an increase in the charge with the increasing upper potential limits employed during the synthesis, except for the film synthesized at 1.60 V for PANI–2APPA. For this particular material, the highly resistive behavior observed was a consequence of its over-oxidation. For the other polymers, the electrochemical redox processes resembled those present in PANI, for which the two redox processes corresponded to the transitions from leucoemeraldine to emeraldine and from emeraldine to pernigraniline, although the presence of over-oxidized chains cannot be ruled out. Interestingly, the redox processes of the voltammetric profiles depended on the aminophenylphosphonic acid employed. Thus, the two characteristic redox processes were clearly observed for polymers synthesized with 4-APPA, even with an upper potential limit during the synthesis of 1.45 V. However, in the case of films obtained with 2-APPA, the stability region of emeraldine decreased from 1.45 V of synthesis potential.

**Figure 5:**
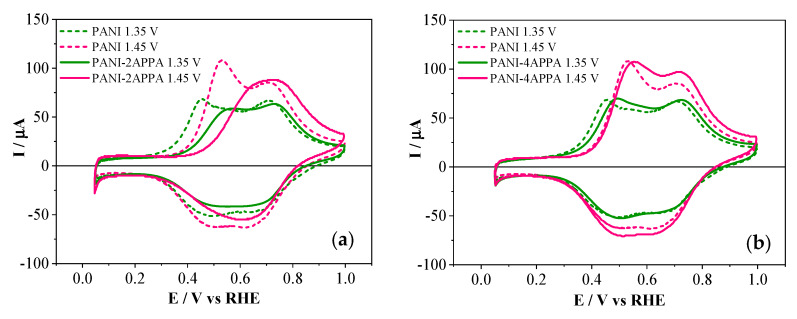
Stable cyclic voltammograms of characterization in 1 M HClO_4_ at 50 mV·s^−1^, obtained after the electrode modification with the copolymer films (monomer concentrations of 1 mM, respectively, at a molar ratio of 1:1): (**a**) PANI–2APPA and (**b**) PANI–4APPA, as well as PANI under the same conditions.

The effect of the phosphorus-containing species in the PANI polymeric chains can be appreciated in Figure 5, where the electrochemical behavior of copolymers and PANI obtained at the same experimental conditions is presented. PANI–2APPA copolymers showed a significant shift of the anodic process associated with the transformation from leuroemeraldine into emeraldine, moving towards more positive potentials compared with PANI. This suggested an impediment of this transition due to the presence on phosphonic groups attached to the polymer backbone [35]. Thus, for 1.35 V the displacement of this first process was about 110 mV, while for 1.45 V a shift of approximately 150 mV was observed. In this sense, some resistance towards oxidation occurred with the incorporation of phosphorus in the polymeric layer. Since there were no new redox processes after copolymerization, the main effect of phosphorus functionalities in the polymeric structure was the displacement of redox processes associated with PANI, making the leucoemeraldine state more stable at higher potentials.

On the other hand, PANI–4APPA copolymers (see Figure 5b) showed an electrochemical response after the synthesis which was quite similar to that of PANI. This may be due to the position of the phosphonic group in the precursor monomer, which hinders its incorporation into the PANI chain. Therefore the copolymer should contain a higher number of aniline rings in relation to the 4-APPA monomer. It should be taken into account that the polymerization of aniline takes place by addition reactions through its *para*-position [38].

The electrochemical behavior observed for both copolymers suggested that the incorporation of the phosphorus groups in the polymer backbone promoted some resistance towards oxidation, making the leucoemeraldine state (a non-conductor state) more stable at higher potentials. Then, a decrease in the conductivity could be expected, as occurs in other substituted polyanilines, for example with sulfonic groups [22].

### 3.3. Spectro-Electrochemical Characterization

**Figure 6:**
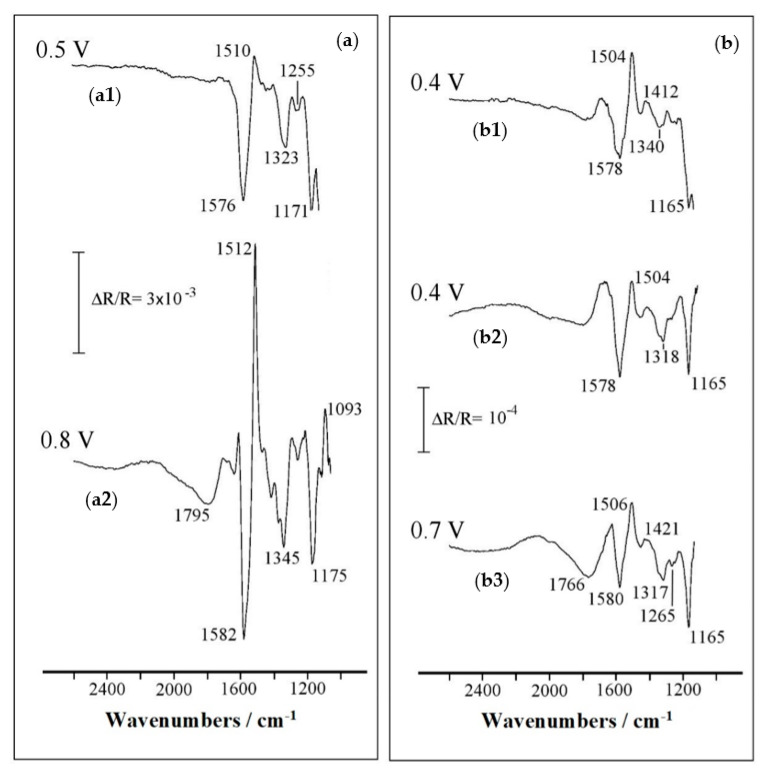
In situ FTIR spectra obtained in 0.1 M HClO_4_ solution for a Pt disc electrode modified with: (**a**) PANI–2APPA synthesized at 1.35 V (**a1**,**a2**) and (**b**) PANI–4APPA synthesized at 1.35 V (**b1**) and 1.45 V (**b2**,**b3**). Monomer molar ratio 1:1, 1 mM. Sample potential is indicated for each spectrum. The reference potential is 0.1 V in all cases. One hundred interferograms were recorded at each potential level. Resolution: 8 cm^−1^.

The characterization of copolymer films by in situ FTIR spectroscopy will increase the knowledge of the chemical species involved in the redox transformations. Figure 6a shows the spectra acquired for PANI–2APPA, previously synthesized at 1.35 V, submitted to applied potentials of 0.5 V and 0.8 V (Figure 6(a1,a2), respectively) (all spectra referred to 0.1 V). The most relevant peak frequencies for the PANI–2APPA spectra are summarized in Table 1, including a proposal of band assignments in both the reduced and oxidized state of the polymer.

The resulting spectra evidenced a similar behavior with respect to PANI (See Figure S8), proving the structural similarity in both materials. Likewise, positively oriented bands at 1510 and 1512 cm^−1^ and negatively oriented bands at 1576 and 1582 cm^−1^ again demonstrated the structural transformation from a benzenoid aromatic ring to a quinoid configuration, respectively. Downward bands were also observed at around 1323–1345 cm^−1^ and 1255 cm^−1^, associated with C-N vibrational modes of amines and imines present in the oxidized structure. The negative band that appeared at 1171–1175 cm^−1^ could be assigned to different contributions, such as C–H bending in the plane and the C-N-C stretching in quinoid units. In addition, this band could be also due to P–C_aromatic_ stretching. Furthermore, the presence of perchlorate anions could not be ruled out from the results obtained, as they could contribute to the previously commented band. They can be inserted into the film to compensate the positive charge generated during the oxidation. A positive band was observed at 1093 cm^−1^ in the spectra at 0.8 V, and could be due to different contributions, including P–O asymmetric stretching [39]. Nevertheless, this band was too close to the lower limit of the frequency range that can be analyzed with a CaF_2_ window, so its origin could not be assured. The P=O stretching band should have been observed at around 1200 cm^−1^ [35,40] but the amine C-N stretching that appeared at around that frequency induced some uncertainty into the assignment.

On the other hand, a new wide negative band at 1795 cm^−1^ appeared at high potentials (0.8 V in the case of PANI–2APPA), potentials that were higher than those of the second peak in the voltammogram (Figure 4a), and whose existence was not observed in PANI. This absorption could be related to active –OH stretching in O=P–OH groups with a single neighboring –OH group. This band was shifted to higher frequencies [41], which could be attributed to interactions between the *ortho*-substituent and the hydrogens in the neighboring ring and to hydrogen bond interactions between phosphorus species and amino groups [16,35].

Figure 6b displays the spectra acquired for PANI–4APPA, previously synthesized at 1.35 V (Figure 6(b1)) and 1.45 V (Figure 6(b2,b3)), submitted to an applied potential of 0.4 V and 0.7 V (reference spectrum at 0.1 V, respectively). In this case, the response showed small differences with respect to PANI or PANI–2APPA, revealing the structural particularities in the copolymer, such as the absence of coupling in *para*-positions between the rings of both monomeric species or a higher branching of the structure induced by a lack of this type of coupling. Moreover, the synthesis at 1.35 V (Figure 6(b2)) or 1.45 V (Figure 6(b2,b3)) did not result in important structural differences at the beginning of the oxidation of the polymer (0.4 V) because the bands observed for both spectra were similar. However, the intensity of the band at around 1766 cm^−1^ increased with the oxidation of the polymer (Figure 6(b3)). It is worth mentioning that this last band appeared at lower frequencies than for PANI–2APPA and could suggest that, in the case of PANI–4APPA, hydrogen bonds within the chain were not favored, but without ruling out the interactions between different polymeric chains. The summary of proposed assignments for the most relevant absorption bands obtained for PANI–4APPA is shown in Table 2.

From the in situ FTIR study of these copolymers it can be concluded that the vibrational changes induced by the applied potential were of the same nature as those of PANI, thus confirming the structural similarity between the new materials and the parent polymer. At this point, a deeper characterization employing techniques such as X-ray photoelectron spectroscopy (XPS) was required to shed more light upon the chemical structures of PANI–2APPA and PANI–4APPA materials.

### 3.4. X-ray Photoelectron Spectroscopy

**Figure 7:**
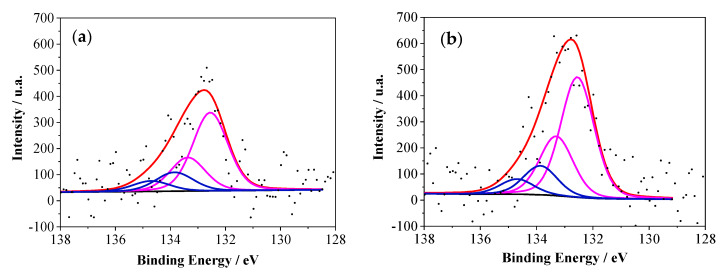
XPS spectra for P2p signals for the copolymers: (**a**) PANI–2APPA and (**b**) PANI–4APPA.

For the XPS characterization, a mirror-polished platinum disk electrode was modified with copolymers deposited at 1.35 V in a working solution that contained the co-monomers at a 1:1 molar ratio and 1 mM concentration. That particular potential was selected because the films showed the best electrochemical behavior. In addition, a pristine PANI film was synthesized under the same experimental conditions and analyzed as a control sample. Figure 7 shows the P2p spectra for the two copolymers. The signal for PANI–2APPA was noisy due to the low amount of P detected. The P2p spectra contained a main peak at around 133 eV that could be deconvoluted into two contributions formed by two asymmetric doublets (Table 3). The first doublet at 132.7 eV was the result of a phosphonic substituent (P^1^) incorporated into the polymer structure, while the second contribution at 133.8 eV was much less intense and may have been associated with phosphoric groups (P^2^) [36,37] generated during synthesis in the oxidative conditions used. The presence of phosphorus species on the polymeric films supported the incorporation of the substituted monomers into the PANI structure, thus generating a true copolymer material.

**Figure 8:**
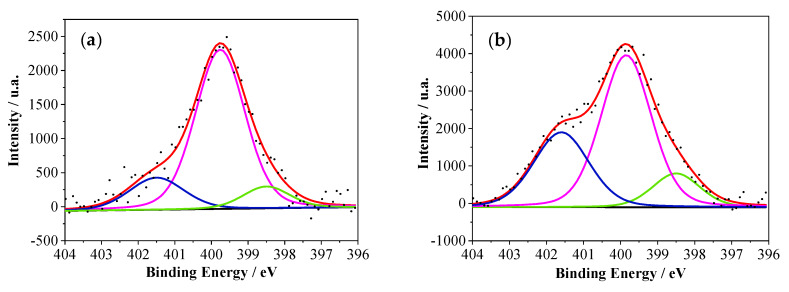
XPS spectra for N1s signals for the copolymers: (**a**) PANI–2APPA and (**b**) PANI–4APPA.

On the other hand, N1s spectra in Figure 8 for PANI–2APPA and PANI–4APPA copolymers could be deconvoluted into three main contributions in all cases, which could be assigned to different nitrogen species. The first and second contributions (N^1^ and N^2^) were observed at lower binding energies at around 398.5 and 399.5 eV, and could be attributed to the presence of imine -type nitrogen (C=N) and amine-type nitrogen (C–N), respectively. These species are usually present in PANI chains (see Figure S9) [18]. The third component (N^3^) appearing at a binding energy close to 401.6 eV could be related to the presence of nitrogen at a higher oxidation state, specifically to positively charged nitrogen in polymeric films generated during electrooxidation [42,45].

Table 3 shows the atomic percentage derived from XPS results for P and N species. As can be observed, the incorporation of phosphorus-containing groups to the PANI structure generated a higher amount of positively charged nitrogen species in comparison to the pristine PANI deposited under the same synthesis conditions. This result, which was more evident for samples modified with 4-APPA, could be attributed to the presence of more terminal-amine groups in the polymer chains, as well as to modifications in the chemical environment induced by phosphonic groups. This phenomenon appeared as a consequence of the self-doping process, which could contribute to stabilizing the positively charged nitrogen species. Regarding C1s spectra (Figure S10), the component related with the presence of C–C (sp^3^) at around 285.5 eV showed an important difference between the copolymers and PANI synthesized under the same conditions. There was a change in the chemical environment that could be associated with the presence of C–P or C–O–P species of phosphonic and phosphoric groups [46] in the PANI structure, promoting a shift towards higher binding energies for copolymers. As expected, this behavior was more pronounced for PANI–4APPA than for PANI–2APPA.

**Figure 9:**
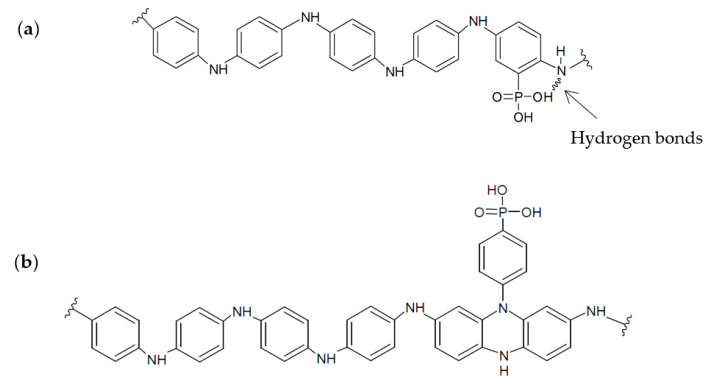
Chemical structures proposed for: (**a**) PANI–2APPA and (**b**) PANI–4APPA copolymers.

According to the quantification obtained from XPS, there was a significant influence of the position of the phosphonic substituent on the modification degree and copolymer growth. Table 3 shows the nitrogen to phosphorus atomic ratio present in the copolymer structure. A lower incorporation of phosphorus into 4-APPA-containing copolymers was detected as compared to those synthesized from 2-APPA. Taking into account that P comes only from 2-APPA or 4-APPA monomers whereas N is present in all of them, it was possible to obtain an APPA:ANI ratio between monomers in the resulting copolymer of 1:4 and 1:6 for PANI–2APPA and PANI–4APPA, respectively. Figure 9 shows proposed structures for these copolymers which are in agreement with these results and those presented above for cyclic voltammetry and in situ FTIR experiments.

The results of chemical composition and atomic ratios obtained by XPS for PANI–4APPA and the electrochemical response (Figure 5b) obtained for this copolymer, similar to those of pure PANI, suggested that the structure of this copolymer was more similar to PANI. The proposed structure (Figure 9) explained the chemical interaction between phosphonic group and the copolymer backbone, as suggested by the in situ FTIR results. On the other hand, the PANI–2APPA copolymer showed a higher APPA/ANI ratio, so a higher amount of APPA monomers was incorporated into the polymer chain. The presence of a higher number of phosphonic groups in the polymer chain (Figure 9) could explain the difficulty in the electron transfer through the polymer chains, which can impede their growth, in accordance with the experimental results. The hydrogen bonds could also be produced between the phosphonic group and the amine group according to in situ FTIR. Moreover, the amount of P present in the polymeric film was lower, but with more of an influence on the electrochemical behavior (see Figure 5a), thus allowing the resistance of PANI to over-oxidation to be modulated, therefore stabilizing the species in its leucoemeraldine redox state. In summary, the copolymerization mechanism of aniline and 2-APPA probably follows the classical head-to-tail coupling found for polyaniline and, as a result, 2-APPA is incorporated to the backbone of the polymer [38]. However, since head-to-tail coupling is impeded for the 4-APPA isomer, this species incorporates as a side group to the growing polymer. In both cases, the formation of inter and intra-chain hydrogen bonds could explain the higher resistance to oxidation.

## 4. Conclusions

The electrochemical oxidation of 2- and 4-aminophenylphosphonic acid (APPA) isomers was studied on platinum electrodes in aqueous acidic medium. Under the experimental conditions employed, homopolymerization was not clearly detected, despite the surface processes characteristic of polycrystalline Pt being blocked.

Phosphonated polyaniline films were successfully deposited on Pt electrodes from the electrochemical copolymerization of aniline with either 2-APPA or 4-APPA. The oxidation up to an upper potential limit of 1.35 V vs RHE was the best condition for the potentiodynamic synthesis of stable polymers, showing significant electroactivity in acidic medium.

The redox transformations of these copolymers and those of PANI were of a similar nature, namely benzenoid-quinoid or amine-imine, among others, as deduced from in situ FTIR results. However, the leucoemeraldine to emeraldine transition in PANI–2APPA copolymer shifted to higher potential values with respect to PANI. In situ FTIR results also suggested that hydrogen bonding occurred between phosphonic and amine groups of this copolymer in a similar way to that described previously for carboxylated polyanilines [16].

In contrast, PANI–4APPA showed an electrochemical behavior that was more similar to that of PANI; this seemed to be related not only to the position of phosphonic groups in the polymer backbone but also to the lower APPA:ANI ratio existing in this material, as deduced from XPS results.

From the APPA monomers it was possible to synthesize polymeric materials with significant amounts of phosphorus in their structure in a controlled manner through copolymerization with PANI.

## Figures and Tables

**Table 1:** Vibrational frequencies and assignments proposed for the reduced and oxidized form of the PANI–2APPA copolymer (molar ratio 1:1, 1 mM) in acidic medium at 0.5 V/0.8 V.

Oxidation State	Frequency/cm^−1^	Assignment	References
**Reduced**	1510, 1512	Benzenoid aromatic ring (C–C) stretching	[41,42,43]
	1300–1310	Secondary aromatic amine (N–H) stretching	[42]
	1220	(P=O) stretching	[35,40]
	1093	(P–O) asymmetric stretching	[39,41]
**Oxidized**	1795	(–OH) stretching in (O=P–OH) with a (–OH) single neighboring	[35,41]
	1576, 1582	Quinoid ring (C–C) stretching	[42,43]
	1323, 1345	Intermediate order (C=N) stretching	[42]
	1255	(C–N^•+^) stretching	[42]
	1171, 1175	(C-H) bending, quinoid ring (C–N–C) stretching, aromatic ring (P–C) stretching and/or ${\mathrm{ClO}}_{4}^{-}$	[40,41,42,44]

**Table 2:** Vibrational frequencies and assignments proposed for the reduced and oxidized form of PANI–4APPA in acidic medium.

Oxidation State	Frequency/cm^−1^		Assignment	References
	1.35 V	1.45 V		
**Reduced**	1504	1504, 1506	Benzenoid aromatic ring (C–C) stretching	[41,42,43]
	1300–1310	1300–1310	Secondary aromatic amines (N–H) stretching	[42]
	1220	1220	(P=O) stretching	[35,40]
**Oxidized**	-	1766	(–OH) stretching in (O=P–OH) with a (–OH) single neighboring	[35,41]
	1578	1578, 1580	Quinoid ring (C–C) stretching	[42,43]
	1340	1317–1318	Intermediate order (C=N) stretching	[42]
	-	1265	(C–N^•+^) stretching	[42]
	1165	1165	(C–H) bending, quinoid ring (C–N–C) stretching, aromatic ring (P–C) stretching and/or ${\mathrm{ClO}}_{4}^{-}$	[41,42,43,44]

**Table 3:** Chemical composition and atomic ratios obtained from the XPS spectra of PANI, PANI–2APPA, and PANI–4APPA samples. Percentages refer to the respective atomic total.

Samples	%N^1^	%N^2^	%N^3^	%P^1^	%P^2^	N/P
**PANI**	19	73	8	-	-	0
**PANI–2APPA**	16	74	10	78	22	5.2
**PANI–4APPA**	12	57	31	80	20	7.5

## Supplementary Materials

The following are available online at https://www.mdpi.com/2073-4360/12/5/1029/s1. Figure S1: Chemical structure of phosphonate monomers: (a) 2-APPA and (b) 4-APPA. Figure S2: Cyclic voltammograms of the open stepwise upper potential limit for the polycrystalline Pt electrode in presence of: (a) 1 mM 2-APPA and (b) 1 mM 4-APPA, in 1 M HClO_4_ at 50 mV·s^−1^ under N_2_ atmosphere. Figure S3: Stable cyclic voltammogram for a platinum electrode in 1 M HClO_4_ at 50 mV·s^−1^. Inset: Picture of a clean platinum electrode. Figure S4: Cyclic voltammograms for synthesis of PANI, obtained during 100 cycles in 1 M HClO_4_ on the polycrystalline Pt electrode at 50 mV·s^−1^ under N_2_ atmosphere at 1.35 V (left) and 1.45 V (right), in the presence of different concentrations of aniline: (a,b) 1 mM, (c,d) 3 mM, and (e,f) 10 mM. Figure S5: Stable cyclic voltammograms of characterization in 1 M HClO_4_ at 50 mV·s^−1^ of PANI previously obtained at different concentrations of aniline during 100 cycles under N_2_ atmosphere at different potentials: (a) 1.35 V and (b) 1.45 V. Figure S6: Cyclic voltammograms of the electrooxidation during 100 cycles in 1 M HClO_4_ on the polycrystalline Pt electrode in the presence of 10 mM 2-APPA at 50 mV·s^−1^ under N_2_ at different potentials: (a) 1.35 V and (b) 1.45 V. Inset: Magnification of the redox processes that occur on the electrode surface during the electrooxidation. (c) Stable voltammograms of the modified electrode in acid media in the absence of the monomer in the solution. Figure S7: Pictures of the modified polycrystalline platinum electrode with the polymeric film of PANI–2APPA (left) and PANI–4APPA (right) deposited onto the surface after 100 cycles at different upper potential limits: (a,b) 1.25 V, (c,d) 1.35 V, (e,f) 1.45 V, and (g,h) 1.60 V. Figure S8: In situ FTIR spectra obtained in 0.1 M H_2_SO_4_ solution of the polycrystalline Pt electrode modified with polyaniline (PANI). Reference spectra acquired at 0.1 V and sample spectra at 0.6 V, 100 interferograms recorded at each potential. Resolution 8 cm^−1^. Table S1: Vibrational frequencies and assignments proposed for the reduced and oxidized form of PANI in acidic medium at 0.6 V. Figure S9: XPS spectra for N1s signals for PANI synthesized at 1.35 V. Figure S10: XPS spectra for C1s signals for PANI, PANI–2APPA, and PANI4–APPA, respectively, synthesized at 1.35 V.
