# Supplementary material for: Synthesis of Phosphorus-Containing Polyanilines by Electrochemical Copolymerization
Source: Polymers (Basel). 2020 May 1;12(5):1029. doi: 10.3390/polym12051029 (PMC7285184; doi:10.3390/polym12051029)
Supplement: Supplementary file 1 [file polymers-12-01029-s001.pdf]

Supplementary Materials

Synthesis of Phosphorus-Containing Polyanilines by Electrochemical Copolymerization

Beatriz Martínez-Sánchez [1], Andrés Felipe Quintero-Jaime [1], Francisco Huerta [2], Diego Cazorla-Amorós [3,*] and Emilia Morallón [1,*]

[1] Departamento de Química Física and Instituto Universitario de Materiales de Alicante (IUMA), University of Alicante, Ap. 99, 03080 Alicante, Spain; beatriz.ms@ua.es (B.M.-S.); andres.quintero@ua.es (A.F.Q.-J.)

[2] Departamento de Ingeniería Textil y Papelera, Universitat Politècnica de València, Plaza Ferrándiz y Carbonell, 1. E-03801 Alcoy, Spain; frahuear@txp.upv.es

[3] Departamento de Química Inorgánica and Instituto Universitario de Materiales de Alicante (IUMA), University of Alicante, Ap. 99, 03080 Alicante, Spain

* Correspondence: cazorla@ua.es (D.C.-A.); morallon@ua.es (E.M.)

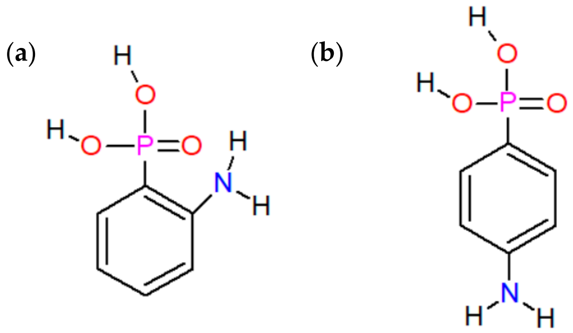

Figure S1. Chemical structure of phosphonated monomers: (**a**) 2-APPA and (**b**) 4-APPA.

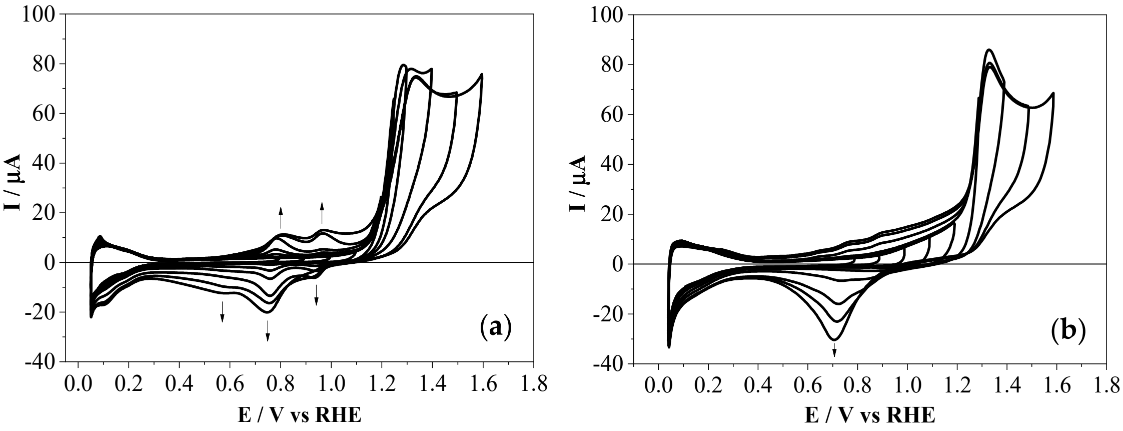

Figure S2. Cyclic voltammograms of the open stepwise upper potential limit for the polycrystalline Pt electrode in presence of: (**a**) 1 mM 2-APPA and (**b**) 1 mM 4-APPA, in 1 M $HClO_4$ at 50 $mV \cdot s^{-1}$ under N_2 atmosphere.

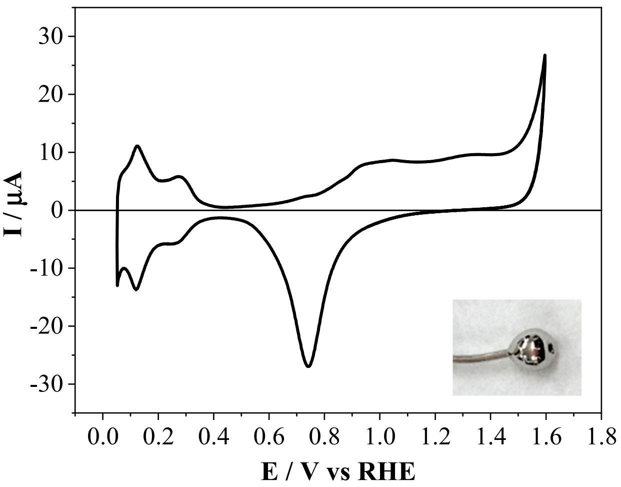

Figure S3. Stable cyclic voltammogram for a platinum electrode in 1 M $HClO_4$ at 50 mV·s^{-1}. Inset: Picture of clean platinum electrode.

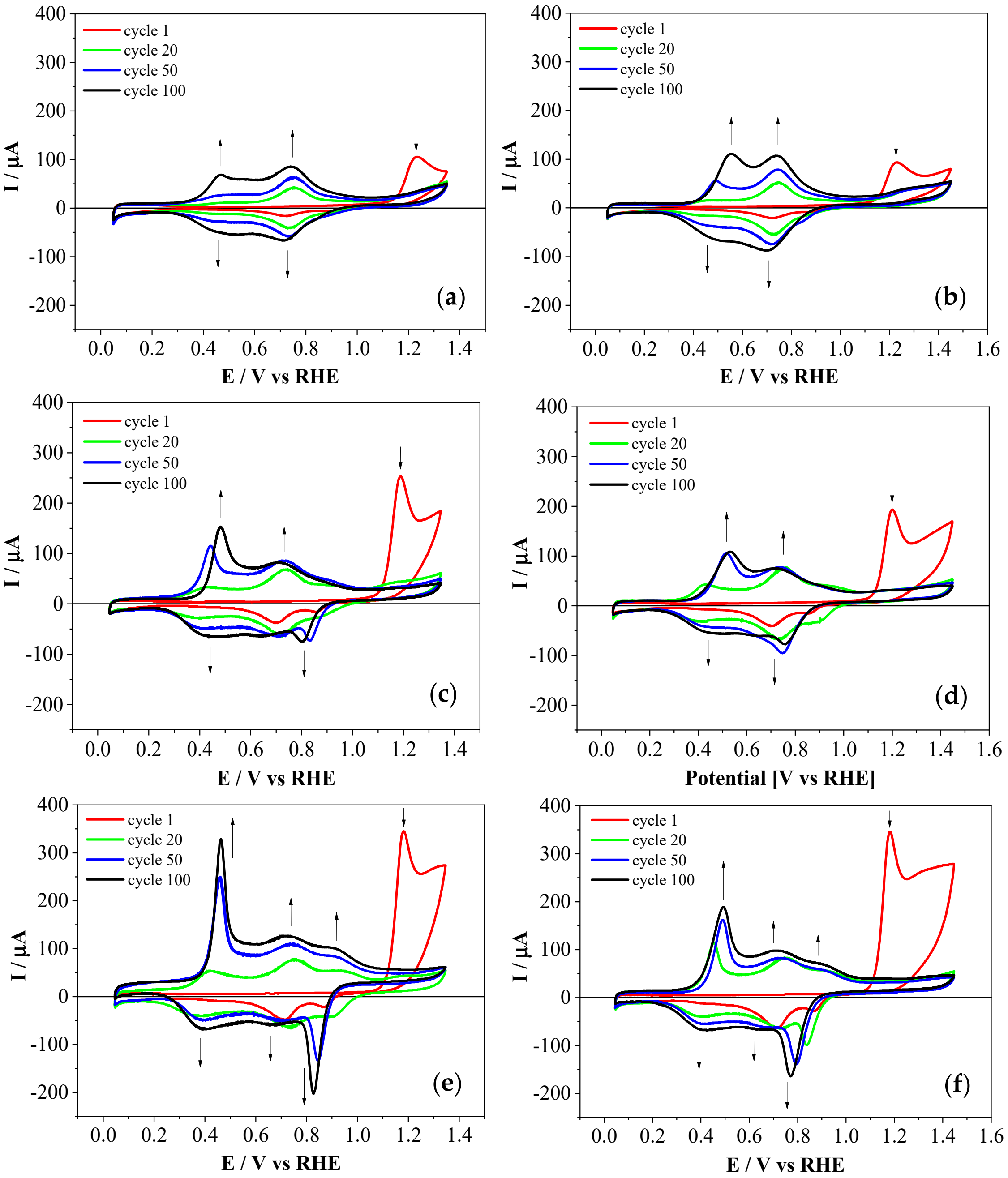

Figure S4. Cyclic voltammograms for synthesis of PANI, obtained during 100 cycles in 1 M $HClO_4$ on the polycrystalline Pt electrode at 50 mV·s^{-1} under N_2 atmosphere at 1.35 V (left) and 1.45 V (right), in presence of different concentrations of aniline: (**a, b**) 1 mM, (**c, d**) 3 mM and (**e, f**) 10 mM.

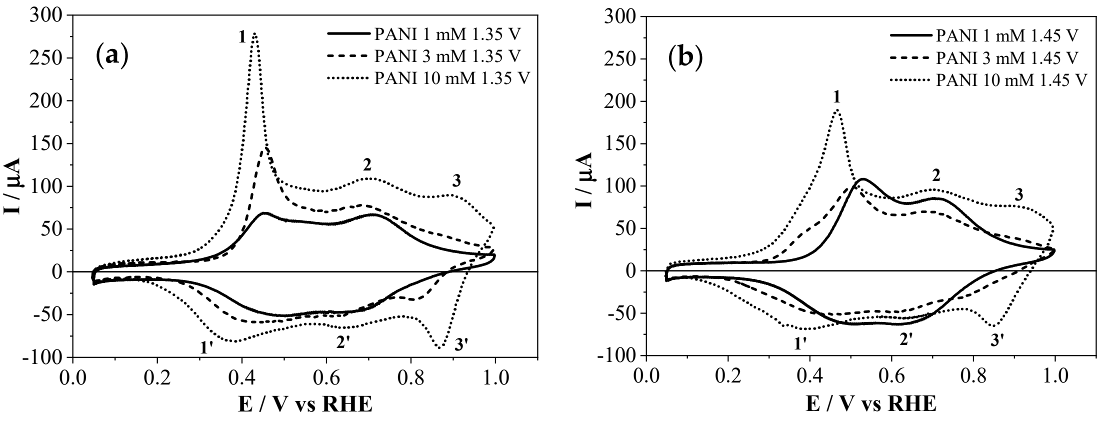

Figure S5. Stable cyclic voltammograms of characterization in 1 M $HClO_4$ at 50 mV·s^{-1} of PANI previously obtained at different concentrations of aniline during 100 cycles under N_2 atmosphere at different potentials: (**a**) 1.35 V and (**b**) 1.45 V.

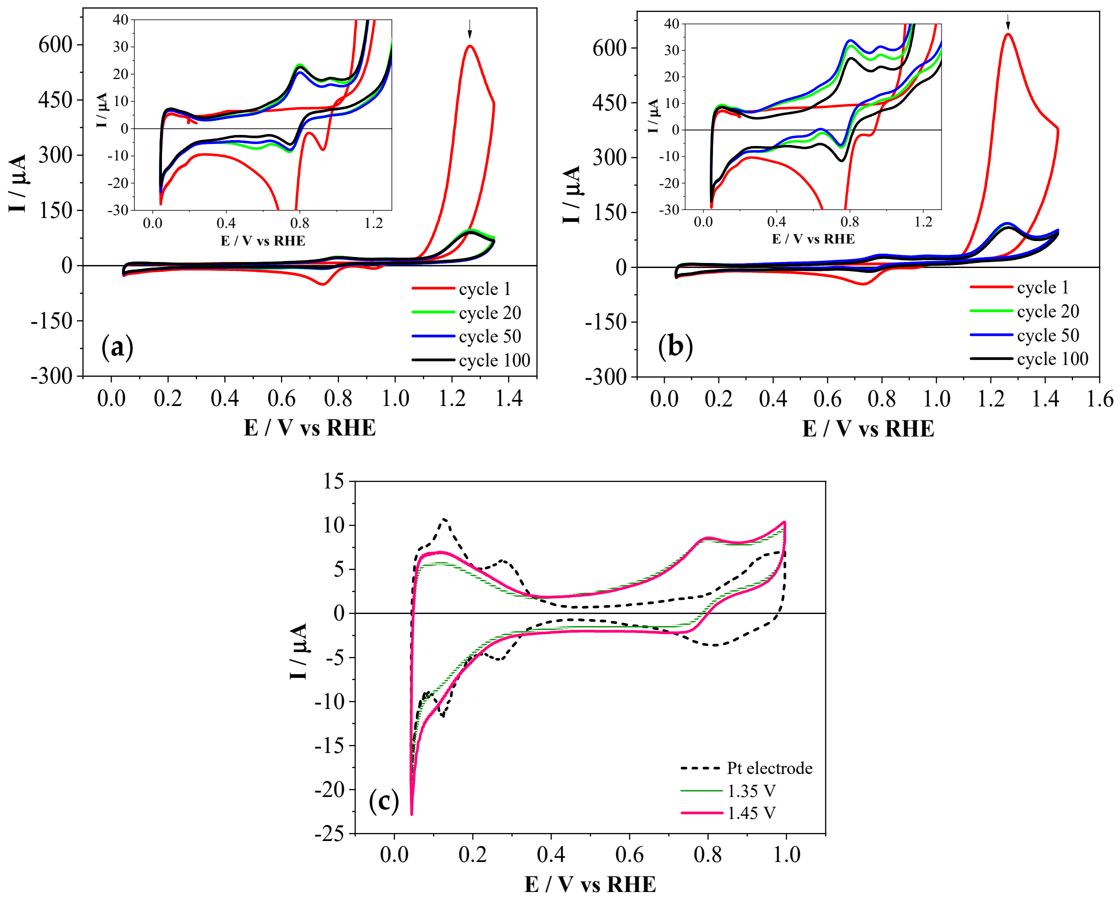

Figure S6. Cyclic voltammograms of the electrooxidation during 100 cycles in 1 M $HClO_4$ on the polycrystalline Pt electrode in presence of 10 mM 2-APPA at 50 mV·s^{-1} under N_2 at different potentials: (**a**) 1.35 V and (**b**) 1.45 V. Inset: Magnification of the redox processes that occur on the electrode surface during the electrooxidation. (**c**) Stable voltammograms of the modified electrode in acid media in absence of the monomer in the solution.

(**a, b**)

(**c, d**)

(**e, f**)

(**g, h**)

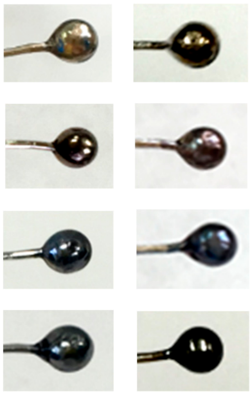

Figure S7. Pictures of the modified polycrystalline platinum electrode with the polymeric film of PANI-2APPA (left) and PANI-4APPA (right) deposited onto the surface after 100 cycles at different upper potential limits: (**a, b**) 1.25 V, (**c, d**) 1.35 V, (**e, f**) 1.45 V and (**g, h**) 1.60 V.

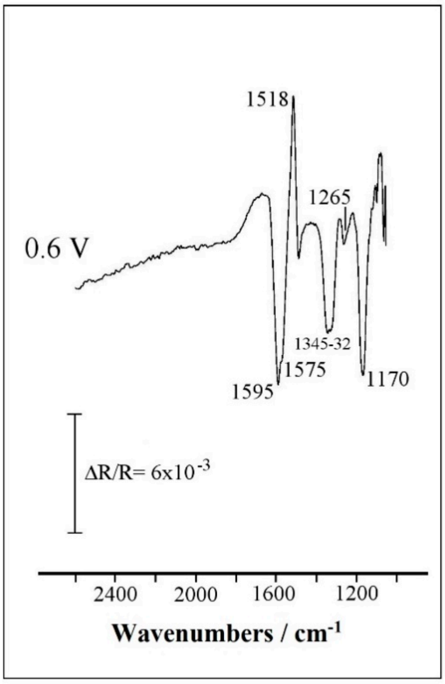

Figure S8. In situ FTIR spectra obtained in 0.1 M H_2SO_4 solution of the polycrystalline Pt electrode modified with polyaniline (PANI). Reference spectra acquired at 0.1 V and sample spectra at 0.6 V, 100 interferograms recorded at each potential. 8 cm^{-1} resolution.

Table S1. Vibrational frequencies and assignments proposed for the reduced and oxidized form of PANI in acidic medium at 0.6 V.

Oxidation State	Frequency / cm^{-1}	Assignments	References
**Reduced**	1518	Benzenoid aromatic ring (C–C) stretching	[41–43]
	1300–1310	Secondary aromatic amines (N–H) stretching	[42]
**Oxidized**	1595	Imine (N-H) bending (>C=N–H) and/or (C=N) stretching	[40,41]
	1575, 1595	Quinoid ring (C-C) stretching	[42,43]
	1332–1345	Intermediate order (C=N) stretching	[42]
	1265	($C–N^{•+}$) stretching	[42]
	1170	(C–H) bending and/or quinoid ring (C–N–C) stretching	[40,44]

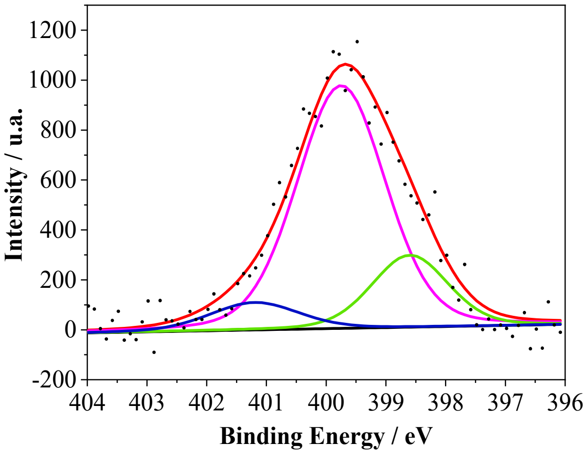

Figure S9. XPS spectra for N1s signals for PANI synthesized at 1.35 V.

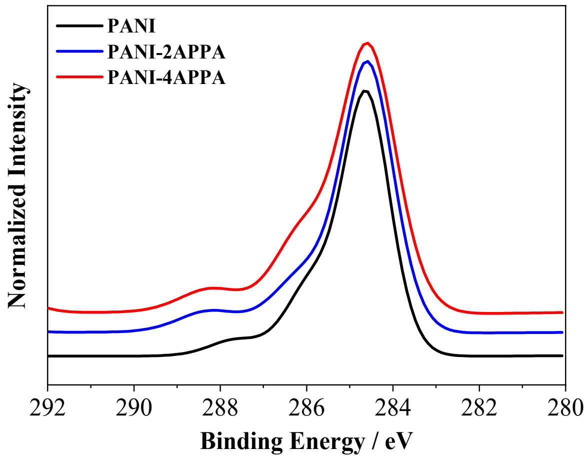

Figure S10. XPS spectras for C1s signals for PANI, PANI-2APPA and PANI-4APPA, respectively, synthesized at 1.35 V.
